# Treatment of Soft-Tissue Necrosis of the Pyriform Sinus Using Pentoxyllifylline and Tocopherol

**DOI:** 10.7759/cureus.19234

**Published:** 2021-11-03

**Authors:** Katie Oxford, Aileen Feschuk, Jamie Tibbo

**Affiliations:** 1 Faculty of Medicine, Memorial University of Newfoundland, St. John's, CAN

**Keywords:** tocopherol, pentoxyllifylline, pento, osteoradionecrosis, oropharyngeal cancer, pentoclo, soft tissue necrosis, head and neck cancer

## Abstract

Radiotherapy of the head and neck can often lead to complications and side effects, including osteoradionecrosis and soft tissue necrosis. One relatively well-established method of treating osteoradionecrosis includes the PENTOCLO protocol, which consists of Pentoxyllifylline, Tocopherol, and Clodronate. Despite its success in the treatment of osteoradionecrosis, the effectiveness of components of the PENTOCLO protocol in treating soft tissue necrosis of the head and neck is underexplored. This case study reviews the successful treatment of a pyriform sinus ulcer that developed after the use of radiotherapy in treating a T3N2b squamous cell carcinoma. The treatment plan used Pentoxyllifylline and Tocopherol, and omitted Clodronate, and can therefore be referred to as a PENTO protocol.

## Introduction

Approximately 50%-60% of patients with head and neck cancer require radiotherapy [1], and it is estimated that complications arise from radiotherapy in 4% to 37% of cases [2]. Osteoradionecrosis is a significant complication that can be treated pharmacologically using pentoxifylline (PTX), tocopherol (vitamin E), and clodronate (together, “PENTOCLO”) [3].

Pentoxifylline is a methylxanthine derivative that improves blood flow through vasodilation and by increasing erythrocyte flexibility [3]. The propensity for radiotherapy to cause intimal proliferation and narrowing of small blood vessels makes increased flexibility of erythrocytes valuable [4]. PTX also possesses an anti-tumor necrosis factor (TNF)-alpha effect which helps to reduce the cytokine cascade [3]. In addition, PTX reduces blood viscosity, increases collagenase activity, and inhibits the proliferation of dermal fibroblasts [5]. Finally, PTX inhibits platelet aggregation and stimulates thrombolysis [4].

Tocopherol alpha (vitamin E) is believed to scavenge reactive oxygen species that are produced under oxidative stress and are implicated in the pathogenesis of osteoradionecrosis [5]. Vitamin E and PTX work synergistically to significantly reduce radiation-induced fibrosis through inhibition of intracellular signaling in response to connective tissue growth factor and TGF-B [5].

Clodronate is a new generation of non-nitrogenous bisphosphonate that has been shown to reduce the number and activity of osteoclasts, thereby inhibiting bone resorption [3].

As mentioned, the most common use of the PENTOCLO protocol in head and neck cancer treatment is for osteoradionecrosis [5]. The use of the PENTOCLO protocol for other head and neck radiotherapy-related complications, specifically soft-tissue radionecrosis, is understudied.

There is a lack of data, surrounding the use of the PENTOCLO protocol in the treatment of radiation-induced soft-tissue necrosis of the head and neck. This case report describes the use of a modified PENTOCLO protocol for the treatment of oropharyngeal soft-tissue radionecrosis. This modified protocol involves the use of Pentoxyllifylline and Tocopherol and omits the use of Clodronate, and can therefore be referred to as a PENTO protocol.

Informed consent was obtained from the patient prior to writing this manuscript.

This article was previously displayed as a poster at the 75th Annual Canadian Society of Otolaryngology Meeting, September 17-19, 2021.

## Case presentation

A 64-year-old male noticed increasing dysphagia, odynophagia, and otalgia with swallowing, approximately six months after completion of concurrent chemotherapy and radiotherapy for the treatment of a T3N2b squamous cell carcinoma (SCC) of the oropharynx. There was also intermittent radiating pain on the left side of the neck, consistent with trigeminal nerve distribution. Other past medical history was deemed noncontributory. The patient was on the following medications: lactulose, allopurinol, vitamin D, omega 3, glucosamine, omeprazole, diclofenac/misoprostol, rosuvastatin, and bisoprolol.

Using flexible nasolaryngoscopy (FNL), an ulcerated area was identified in the left pharyngeal wall. The area was biopsied on two separate occasions, and no malignancy was identified either time. The absence of biopsy-proven cancer led to a presumed diagnosis of soft-tissue radionecrosis. As an alternative to classical treatment options, such as hyperbaric oxygen therapy (HBOT) [6], a PENTO protocol was proposed after a multidisciplinary cancer board determined that HBOT may present a risk to this particular patient. With this in mind, the patient chose to pursue a PENTO protocol. 

The PENTO protocol was utilized as follows: PTX (pentoxifylline) 400 mg PO BID, vitamin E 500 IU BID, prednisone 20 mg PO OD (two days/week, Sat-Sun), Ciprofloxacin 1,000 mg PO (two days/week, Sat-Sun).

The decision to exclude clodronate, one of the typical components of the PENTOCLO protocol, from the treatment plan was made based on the fact that, unlike for osteoradionecrosis, the bone preserving qualities of clodronate were not deemed useful for soft-tissue necrosis. This modification of the PENTOCLO protocol, and its use for soft-tissue necrosis rather than osteoradionecrosis, sets this case apart from others.

**Figure 1:**
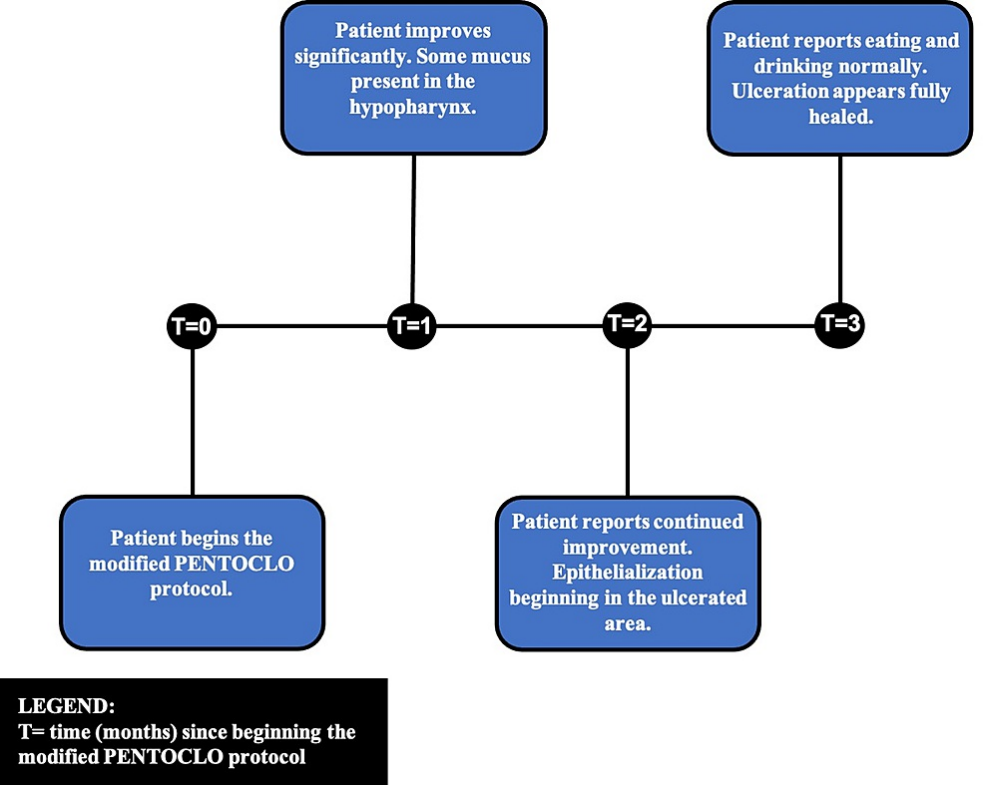
Timeline of patient recovery from soft-tissue necrosis of the oropharynx on the PENTO protocol.

Within one month of being on the PENTO protocol, examination of the oral cavity and oropharynx revealed some improvement in the ulcerated area. After two months of being on this treatment plan, the patient was doing very well, and reported significant improvement in his eating abilities, with little throat discomfort. Examination using FNL showed epithelialization of the ulcerated area had begun. After three months of being on the PENTO protocol, it was noted that the patient was doing extremely well. The patient was reportedly eating and drinking normally with no restrictions, and weight gain was noted. On subsequent FNL, the ulcerated lesion appeared to have fully healed. However, the patient was kept on the PENTO protocol for an additional month to ensure success. The timeline of this patient’s recovery on the PENTO protocol can be visualized in Figure 1.

## Discussion

Soft-tissue necrosis can be a debilitating adverse effect of radiation therapy, introducing a negative impact on patient quality of life [7]. When osteoradionecrosis is managed conservatively, the use of hyperbaric oxygen can be successful in 25%-44% of cases [8]. A review focusing on soft tissue necrosis of the larynx found a response rate of 82% when patients were treated with hyperbaric oxygen [6].

It is important to note that hyperbaric oxygen is not without adverse effects [9]. Some adverse effects include middle ear barotrauma, sinus/paranasal barotrauma, air-gas embolism, hyperoxic myopia, and oxygen toxicity seizures [9]. Claustrophobia can also become an issue for patients during their treatment [9]. In addition, access to hyperbaric oxygen may be variable, depending on geographical location, hospital wait times, and patient ability to engage in long-term treatment. The administration of HBOT may differ by the institution; however, generally, the treatment is administered for 1-2 hours daily for between 30 and 60 treatments [10]. This may be a difficult regimen for some patients to adhere to.

In a study performed by Dissard et al., the medications involved in the PENTO protocol were shown to be tolerated very well by patients [11]. No severe adverse events were reported [11]. Non-severe adverse events documented include: “diarrhea (22.2%), epigastralgia (11.1%), asthenia (11.1%), nausea (7.4%), and insomnia (3.7%)” [11]. The use of the PENTO protocol has potential as an alternative treatment of soft tissue radionecrosis, especially in special circumstances when hyperbaric oxygen may not be well tolerated by a patient.

The use of a PENTO protocol for the treatment of soft-tissue necrosis is highly underexplored. However, a systematic review/meta-analysis of the use of PENTO for the treatment of osteoradionecrosis was published in 2018 [12]. Of the 211 patients with osteoradionecrosis treated with PENTO, 127 fully recovered or significantly improved and did not require further intervention [12]. Sixty patients' osteoradionecrosis neither improved nor deteriorated while on PENTO [12]. Ten patients were lost to follow-up and 15 patients experienced disease progression while on PENTO [12]. It is important to note that the logic to exclude clodronate in the treatment of the patient discussed in this case study (i.e. because soft tissue necrosis would not benefit from the bone preserving effects of clodronate) does not hold true for the patients included in the systematic review (who were suffering from osteoradionecrosis). Therefore, the discussion of this systematic review was for the purpose of investigating the use of PENTO in general, but it is important to note that the systematic review and our case study cannot be compared seamlessly.

Although promising, it is important to note that this is a single case report, and no randomized clinical trials using PENTO for soft-tissue radionecrosis treatment have been performed. Therefore, this case report must be interpreted with adequate caution. These findings suggest that randomized controlled trials should be pursued in the future, as there may be potential for positive outcomes for patients experiencing soft tissue radionecrosis. It is our hope that this case study provokes conversation about the potential of the PENTO protocol, and leads to further evaluation of its efficacy as a treatment modality for post-radiation soft tissue necrosis.

## Conclusions

Soft-tissue radionecrosis in the context of head and neck cancer is a significant complication that drastically affects patients’ quality of life. Currently, research in this area is minimal and preliminary. To our knowledge, no randomized control trials have been conducted, leaving a gap in the literature and a gap in patient care. This case report suggests that a PENTO protocol, consisting of Pentoxyllifylline and Tocopherol, may be useful in the treatment of soft-tissue radionecrosis, and opens the door for more research to investigate this possibility. Patients with soft-tissue radionecrosis may benefit from a significantly improved quality of life with the use of this non-invasive medical therapy. It is critical that further research be done in this area in order to determine the efficacy of this treatment.
